# Analysis of cerebrospinal fluid from cattle with central nervous system disorders after storage for 24 hours with autologous serum

**DOI:** 10.1186/s12917-015-0502-x

**Published:** 2015-08-13

**Authors:** C. Bellino, B. Miniscalco, I. Bertone, A. Cagnasso, E. Occhiena, P. Gianella, A. D’Angelo

**Affiliations:** Department of Veterinary Science, University of Turin, Grugliasco (TO), Turin, Italy; Private Practitioner, Turin, Italy

**Keywords:** Cattle, CNS disorders, CSF analysis, 24-h storage

## Abstract

**Background:**

We compared the changes in cell morphology, total and differential cell counts between cerebrospinal fluid (CSF) samples analyzed within an hour of collection (fresh sample) and after the addition of autologous serum and storage for 24 h (stored sample) in 27 cattle with central nervous system disorders.

**Results:**

There was a positive linear correlation between total and differential cell counts in the fresh and the stored samples. Cell morphology was preserved in all stored samples, except for increased vacuolization of mononuclear cells and cleaved nuclei of some small mononuclear cells. In the stored CSF samples, the total nucleated cell count and monocyte percentage were decreased (P = 0.01; P = 0.03), while the lymphocyte percentage was increased (P = 0.04). Mononuclear pleocytosis diagnosed in 20 fresh samples was cytologically confirmed in 12 of the 20 stored samples. In the remaining eight stored samples, the number of total nucleated cells was within the normal range. Neutrophilic pleocytosis was confirmed in all seven stored samples.

The overall agreement rate between cytologic interpretation of the fresh and the stored CSF samples was 70 % (100 % for neutrophilic pleocytosis and 60 % for mononuclear pleocytosis).

**Conclusions:**

Adding 11 % of autologous serum to CSF samples might allow delayed analysis with a good agreement rate for CSF cytological interpretation. Caution is nonetheless warranted, as animal age, anamnesis, and neurological presentation need to be considered when interpreting stored CSF without pleocytosis.

**Electronic supplementary material:**

The online version of this article (doi:10.1186/s12917-015-0502-x) contains supplementary material, which is available to authorized users.

## Background

Cerebrospinal fluid (CSF), formed by plasma ultrafiltration and active transport mechanism of substances across the blood–brain barrier, circulates in the subarachnoid space of the entire central nervous system (CNS) [1–5]. Besides its protective function toward the CNS, CSF serves to transport neurotransmitters, maintain ionic balance, and excrete cerebral metabolites [3–6].

CFS analysis can be a useful tool in the diagnosis of neurological diseases in living animals [7–9] and can aid in narrowing differential diagnosis of inflammatory, degenerative or neoplastic disorders [10]. Moreover, differential cell count and cellular morphology can help to better define pathological processes [11].

With adequate restraint, CSF samples can be safely collected from either the cerebellomedullary cistern or the lumbosacral site in ruminants [3, 4, 9]. Because of time and temperature-dependent cell deterioration, which may preclude correct interpretation of findings, CSF samples should be submitted for analysis within 30 to 60 min after collection [4, 10, 12]. The diagnostic life span of CSF samples can be extended by adding either hydroxyethyl starch (hetastarch) [12], formalin or ethanol [13] or preparing a cytoconcentrate with a sedimentation device [14]. The latter technique, however, is impractical for field testing in cattle practice. The addition of autologous or fetal serum to aliquots of feline and canine CSF samples has been shown to preserve cells and stabilize the specimen for up to 24 h [10, 12]. In a previous study on CSF from healthy calves, we found no significant differences between the total and differential cell counts in the fresh and the stored CSF samples, added with 11 % autologous serum and then stored for 24 h at 4 °C [15]. The slight differences between the stored and the fresh CSF samples did not affect the diagnostic clinical interpretation [15].

Building on the results of this previous work [15], we evaluated the changes in cell morphology, total and differential cell counts between CSF samples analyzed within 1 h of collection (fresh sample) and after the addition of autologous serum and storage for 24 h (stored sample) in 27 cattle with central nervous system disorders.

## Methods

This prospective study was conducted in accordance with current animal welfare regulations (Directive 98/58/EC and Italian Decree Law 146/2001). Cattle referred to the Teaching Hospital of the Department of Veterinary Science of Turin (Italy) for a neurological problem between March 2009 and March 2013 were selected for the study following physical and neurological examination, the latter by a board-certified neurologist (ADA). Complete blood count (CBC), biochemical profile (aspartate aminotransferase [AST], alkaline phosphatase [ALP], blood urea nitrogen [BUN], creatinine, glucose, creatine phosphokinase [CPK], total serum protein, albumin, calcium [Ca], magnesium [Mg] and phosphorus [P]) and CSF analysis were performed. In selected cases, blood gas analysis, coagulation profile, bacterial culture, synovial fluid analysis, cytologic evaluation, leukocyte immunophenotyping and postmortem examination were additionally performed to establish diagnosis. Only samples with a total cell count >10 cells/mm^3^ were included in the study.

### Collection of Blood and CSF

Blood samples (5 mL aliquots) were collected from the jugular vein into serum and EDTA vacuum tubes. Serum was separated by centrifugation at 1500 *g* for 10 min. CSF was aseptically collected under sedation from the lumbosacral site while the animal was standing, as previously described [15]. Each sample was divided into two equal aliquots, one of which was analyzed within 1 h of collection (defined as fresh sample). Autologous serum was added to the second aliquot to a final concentration of 11 % and stored for 24 h at 4 °C (defined as stored sample).

### CSF analysis

For the total nucleated cell count, 100 μL CSF was added to 100 μL Turk stain and placed on a Nageotte hemocytometer, and the number of cells in an eight by 10 rectangle was counted. Two slides, one for a differential cell count and one for morphological evaluation, were prepared by cytocentrifugation at 500 *g* for 5 min (Cytospin2, Shandon) of 250 μL CSF and stained with May-Grünwald Giemsa. Only samples with at least 10 cells per slide were used for the differential cell count. The percentages of neutrophils, eosinophils and mononuclear cells (lymphocytes and monocytes) were calculated [14]. Total protein concentration of CSF was measured by spectrophotometry (ILab Aries, Instrumental Laboratory) using a pyrogallol red assay only on the fresh samples. Cellular morphology, total and differential cell counts of the stored CSF samples were analyzed in the same way and by the same cytologist (BM) 24 h later.

CSF samples were considered abnormal if the total cell count was >10 cells/ mm^3^ and/or the protein content was >400 mg/L. Only samples with total and differential cell counts before and after storage were included.

### Statistical analysis

Data were analyzed using the R ver. 2. 1.0 freeware statistical software package. The Shapiro-Wilk normality test was used to determine whether the data followed a normal distribution. None of the data was normally distributed. Numerical data are presented as median, minimum and maximum. Not normally distributed data were analyzed using the Wilcoxon rank sum test. Spearman’s rank correlation coefficient (R*s*) was used to test the strength of correlations. Statistical significance was set at *P* <0.05.

## Results

Thirty cattle with neurological disorders from farms in Piedmont were enrolled (mean age, 1 year; range, 4 days – 7 years). The majority of the animals were female (70 %, 21/30) and beef cattle (77 %, 23/30). Piedmontese breed accounted for 91 % (21/23). Other breeds included Italian Holstein-Friesian (n = 5), Blonde d’Aquitaine (n = 2), and crossbreed (n = 2). A definitive neurological diagnosis was available for all animals. An additional file shows this in more detail on the basis of the VITAMIN D algorithm [16] [see Additional file 1].

The most frequent neuroanatomical location of lesions was the forebrain and brainstem (78 %).

In all, 27 out of 30 samples met the inclusion criteria. Total protein concentration in the fresh samples was >400 mg/L in 18 animals. Total nucleated cell count was significantly decreased in the stored CSF samples (P = 0.01). Concerning the differential cell count, monocyte and lymphocyte percentages were significantly decreased (P = 0.03) and increased (P = 0.04) in the stored samples, respectively. No significant differences in neutrophil and mononuclear percentages were found between the fresh and the stored samples (Table 1).

**Table 1:** Protein concentration, total and differential cell counts in CSF samples

Measurement	Fresh CSF	Stored CSF	*P*
	median (min - max)	median (min - max)	
Total protein (mg/L)	769 (184–28,084)	–	NA
Total cells (L)	41.0 × 10^6^ (11.9 × 10^6^–12,310.0 × 10^6^)	20.9 × 10^6^ (2.0 × 10^6^–1,664 × 10^6^)	0.01
Differential cell count			
Neutrophils (%)	4.0 (0.0–97.0)	3.00 (0.0–95.0)	NS
Mononuclear cells (%)	96.0 (3.0–101.0)	97.00 (5.0–100.0)	NS
- Monocytes (%)	52.0 (1.0–96.0)	30.0 (1.0–97.0)	0.03
- Lymphocytes (%)	22.0 (1.0–73.0)	32.0 (1.0–87.0)	0.04

NA- Not Available

NS- Not Significant

Mononuclear pleocytosis was observed in 20 fresh CSF samples and neutrophilic pleocytosis in seven fresh samples. Cytological interpretation of mononuclear pleocytosis was confirmed in 12 out of 20 stored CSF samples. In the remaining eight stored samples, the number of total nucleated cells was within the normal range. The total protein content in three of these eight stored samples was greater than the reference range in the corresponding fresh samples. Neutrophilic pleocytosis was confirmed in all seven stored samples. The overall agreement rate between cytological interpretation of the fresh and the stored CSF samples was 70 % (100 % for neutrophilic pleocytosis and 60 % for mononuclear pleocytosis). There was a positive linear correlation between the total and differential cell counts in the fresh and the stored samples (Table 2). No linear correlation between total protein concentration (serum and CSF) and the degree of cell deterioration was found [CSF protein (P = 0.1; Rs = 0.31); serum protein (P  = 0.67; Rs = 0.1)].

**Table 2:** Coefficients of linear correlation between total and differential cell counts in fresh/stored CSF samples

Measurement	Correlation coefficient (R_s_)	*P*
Total cells (mm^3^)	0.87	<0.0001
Neutrophils (%)	0.90	<0.0001
Mononuclear cells (%)	0.89	<0.0001
Monocytes (%)	0.78	<0.0001
Lymphocytes (%)	0.79	<0.0001

**Fig. 1:**
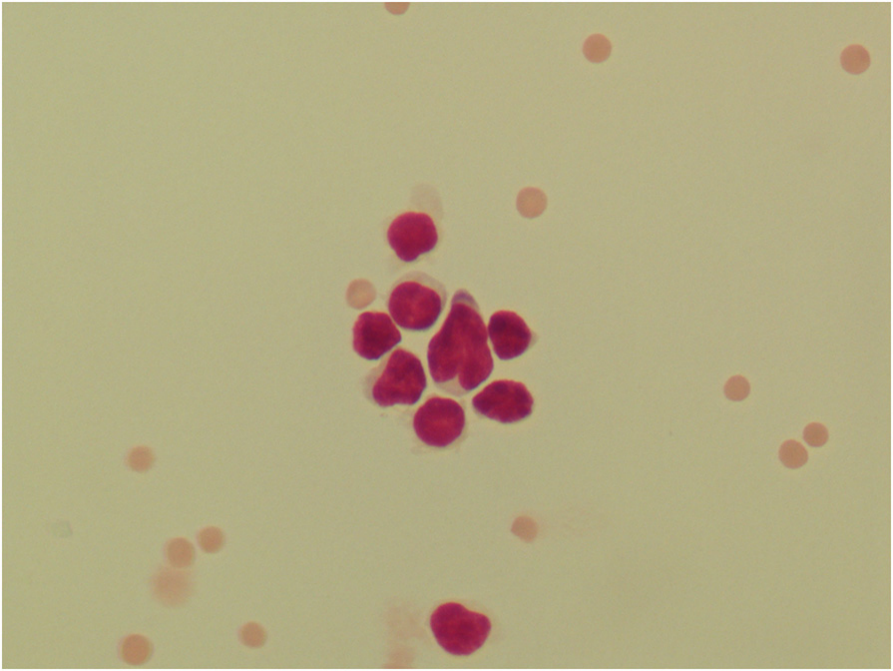
Mononuclear pleocytosis (lymphoma). Fresh sample, May-Grunwald Giemsa (600x)

**Fig. 2:**
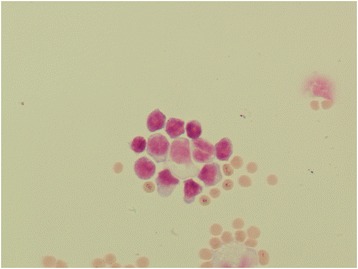
Mononuclear pleocytosis (lymphoma). Stored sample showing preserved cell morphology, except for increased vacuolization of mononuclear cells. It is also present a mononuclear cell with cleaved nuclei, May-Grunwald Giemsa (600x)

**Fig. 3:**
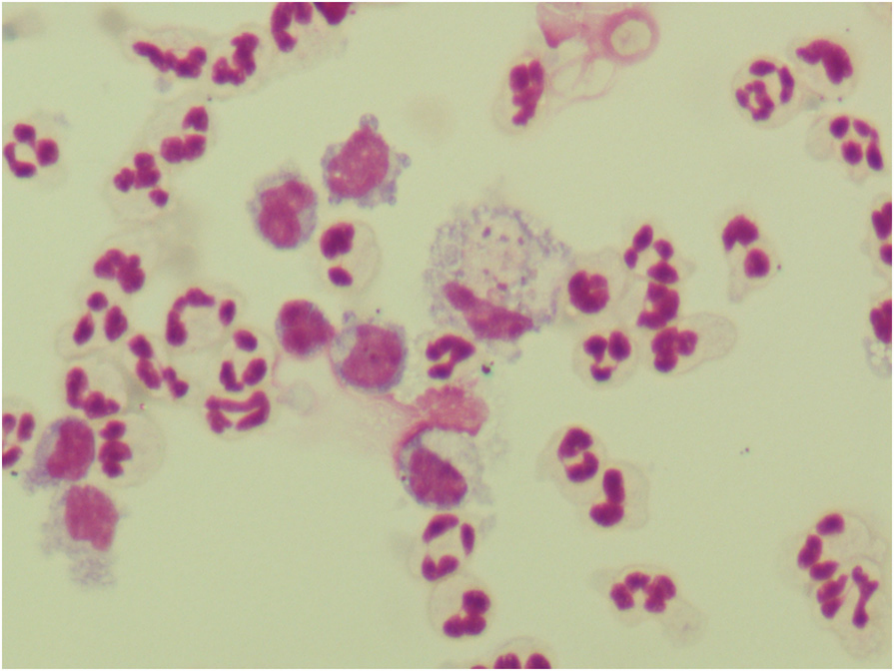
Neutrophilic pleocytosis. Fresh sample, May-Grunwald Giemsa (600x)

**Fig. 4:**
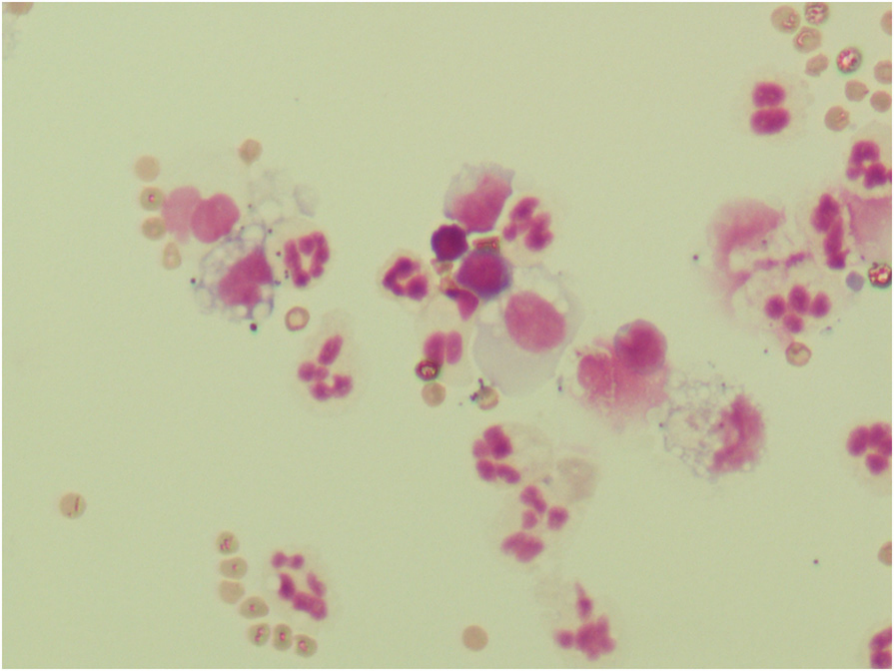
Neutrophilic pleocytosis. Stored sample, showing mononuclear cells with increased vacuolization, May-Grunwald Giemsa (600×)

Cell morphology was preserved in all the stored samples, except for increased vacuolization of mononuclear cells and cleaved nuclei of some small mononuclear cells (Figs. 1, 2, 3, 4).

## Discussion

Cytological interpretation of CSF is key to establishing a clinical diagnosis [8, 9]. A previous study on CSF from dogs and cats reported that even if the number of unrecognizable cells increases after 24 and 48 h of storage, the number of intact cells needed for diagnosis is sufficient and that the diagnostic interpretation of the fresh and the stored CSF samples was the same in both instances [10]. A more recent study on CSF from clinically healthy calves showed that storage time in the presence of 11 % autologous serum did not influence the diagnostic interpretation [15]. Both studies suggest that the storage of CSF added with 11 % of autologous serum does not alter its cytological interpretation in either small or large animals, with or without neurological disorders, respectively. In the present study, the addition of 11 % autologous serum to the CSF samples from cattle with neurological disorders proved to be a valid storage method in the majority of cases (70 % agreement rate). Nevertheless, the total nucleated cell count was within the normal range in eight stored CSF samples. Five of these eight samples were considered normal after 24 h of storage with regard to the number of total nucleated cells, cellular pattern, and morphology. Mononuclear pleocytosis was diagnosed before storage, confirming the hypothesis that mononuclear cells degenerate earlier [12]. In the remaining three stored CSF samples, albuminocytological dissociation was diagnosed based on a normal total leukocyte count and increased total protein concentration in the corresponding fresh samples [13]. This finding, along with anamnestic data and clinical signs, might have suggested a space-occupying lesion such as an abscess or, less frequently in bovine neurology, a hematoma or tumor.

Despite the good linearity between total and differential cell counts in the fresh and the stored samples and despite satisfactory preservation of cell morphology, there was a significant difference between total and differential cell counts before and after storage. This finding contrasts with previous studies in both small and large animals [10, 12, 15]. Indeed, we noted a significant decrease in both total cell count and monocytes and a significant increase in lymphocytes in the stored CSF samples. Since the differential cell count is based on a 100-cell count and the number of cells is expressed in percentage, the decrease in monocytes may have influenced the increase in lymphocytes. This hypothesis is supported by the fact that the percentage of mononuclear cells (monocytes and lymphocytes), though increased, did not differ significantly after storage. The change in the proportion of neutrophils was not statistically significant, supporting the hypothesis that neutrophils degenerate more slowly than mononuclear cells [12]. This finding contrasts, however, with observations from a previous study which reported that neutrophils seem to be more susceptible [10].

In contrast with previous studies [10, 12] no linear correlation between total protein concentration and the degree of cell deterioration was not found. Oncotic pressure or similar factors have been proposed as possible mechanisms of preservation [12]; however, since no attempts were made to elucidate this hypothesis, definitive conclusion can not be drawn.

As reported above, the results of the present study do not completely reflect what we would have expected on the basis of our previous work on healthy cattle nor are they consistent with published data on small animals. This difference is difficult to explain since sample collection methods, storage time, processing and reading (the cytologist was the same) were the same in both studies. We can only assume that many of the animals with neurological problems had been treated with antibiotics, steroids and non-steroidal anti-inflammatory drugs by the attending veterinarian before being referred. If we had had reliable information about their treatment history, we could have created two groups (treated and non-treated) and then attempted to confirm or reject the over mentioned assumption that previous treatments would potentially affect the degree of cell deterioration. Therefore, the lack of complete anamnestic information is the major limitation of this study.

For the purposes of the present study, we used autologous serum rather than other stabilizing agents (e.g., fetal calf serum or hetastarch), as it is easier and more practical to obtain and handle in field conditions.

## Conclusions

Our findings support the recommendation to analyze CSF within 1 h of collection [4, 10, 12]. Since this is not always easy to do in cattle practice, adding 11 % of autologous serum to CSF samples might allow delayed analysis with a good agreement rate for CSF cytological interpretation. Caution is nonetheless warranted, as animal age, anamnesis, and neurological presentation need to be considered when interpreting stored CSF without pleocytosis.

## Additional file

Additional file 1: 
**Classification of neurological diagnosis on the basis of the VITAMIN D algorithm** **[** 16 **].** (XLSX 12 kb)

## Abbreviations

ALP: Alkaline Phosphatase
AST: Aspartate Aminotransferase
BUN: Blood Urea Nitrogen
CBC: Complete Blood count
CCN: Cerebrocortical Necrosis
CNS: Central Nervous System
CPK: Creatine Phosphokinase
CSF: Cerebrospinal Fluid
NA: Not available
NS: Not significant
P: Phosphorus
